# Comparative Metabolic Profiling of Different Colored Rice Grains Reveals the Distribution of Major Active Compounds and Key Secondary Metabolites in Green Rice

**DOI:** 10.3390/foods13121899

**Published:** 2024-06-17

**Authors:** Mingchao Zhao, Linan Zhai, Qingjie Tang, Junfang Ren, Shizhen Zhou, Huijian Wang, Yong Yun, Qingwen Yang, Xiaowei Yan, Funeng Xing, Weihua Qiao

**Affiliations:** 1 Sanya Institute, Hainan Academy of Agricultural Sciences, Sanya 572000, China13518841701@163.com (Q.T.); 13078994838@163.com (X.Y.); 2 Cereal Crops Institute, Hainan Academy of Agricultural Sciences, Haikou 571100, China; 3 National Nanfan Research Institute (Sanya), Chinese Academy of Agricultural Sciences, Sanya 572024, China

**Keywords:** pigmented rice, bioactive compounds, LC–MS, vitamin, metabolomics

## Abstract

Pigmented rice grains are important resources for health and nutritional perspectives. Thus, a thorough dissection of the variation of nutrients and bioactive metabolites in different colored rice is of global interest. This study applied LC–MS-based widely targeted metabolite profiling and unraveled the variability of metabolites and nutraceuticals in long grain/non-glutinous black (BR), red (RR), green (GR), and white rice (WR) grains. We identified and classified 1292 metabolites, including five flavonoid compounds specific to BR. The metabolite profiles of the four rice grains showed significant variation, with 275–543 differentially accumulated metabolites identified. Flavonoid (flavone, flavonol, and anthocyanin) and cofactor biosynthesis were the most differentially regulated pathways among the four rice types. Most bioactive flavonoids, anthocyanidins (glycosylated cyanidins and peonidins), phenolic acids, and lignans had the highest relative content in BR, followed by RR. Most alkaloids, amino acids and derivatives, lipids, and vitamins (B6, B3, B1, nicotinamide, and isonicotinic acid) had higher relative contents in GR than others. Procyanidins (B1, B2, and B3) had the highest relative content in RR. In addition, we identified 25 potential discriminatory biomarkers, including fagomine, which could be used to authenticate GR. Our results show that BR and RR are important materials for medicinal use, while GR is an excellent source of nutrients (amino acids and vitamins) and bioactive alkaloids. Moreover, they provide data resources for the science-based use of different colored rice varieties in diverse industries.

## 1. Introduction

The ever-increasing human demand for healthier foods has stimulated scientists’ interests to understand and modulate the phytochemical profiles of plant-derived foods [1]. Cereals, including wheat, barley, sorghum, rice, maize, and oats, are the most widely consumed foods and represent essential materials for developing functional foods and reducing the incidence of chronic diseases [1,2,3]. Among them, rice is the most important staple food globally (especially in developing countries), with a higher contribution to tackling the global hunger index and achieving food security [4,5]. There are different types of rice depending on the color (white/pigmented), stickiness during cooking (normal/glutinous), and grain size (long/short). Pigmented rice includes those with colors ranging from deep purple to brown-reddish due to the accumulation of natural pigments in the seed coat, pericarp, and aleurone [6]. The grain qualities of pigmented rice are beyond the range of common rice varieties, particularly in terms of their chemical composition, physical characteristics, and aroma [6]. Pigmented rice grains have been used as key ingredients in traditional medicine in Korea, China, and Japan since ancient times to treat anemia and diabetes, enhance kidney function and blood circulation, and relieve blood congestion [7]. Supportively, pharmacological investigations have shown that pigmented rice, especially black and red rice, possess antioxidant, anti-diabetic, anti-hypertension, cardioprotective, anticancer, antiallergic, anti-hyperlipidemia, antitumor, antiatherosclerosis, and protective effects against alcoholic liver disease [8,9,10,11,12,13]. Unfortunately, although there is evidence for the higher nutritional and therapeutic values of pigmented rice compared to white rice, the global metabolome differences between the different colored rice remain not well elucidated, limiting their use in specific dietary formulations and food pharmacy programs.

Pigmented rice grains are rich in a variety of antioxidant compounds, including flavones, flavonols, isoflavones, anthocyanins, procyanidins, phenolics, tannins, tocols, sterols, γ-oryzanols, vitamins, amino acids, and essential oils [5,6,14,15]. Black rice is particularly rich in anthocyanins (cyanidin 3-galactoside, cyanidin 3,5-diglucoside, cyanidin 3-glucoside, cyanidin 3-rutinoside, peonidin 3-glucoside, etc.), whereas red rice contains higher levels of procyanidins [16,17]. Due to their quality values, studies have focused on dissecting the genetic basis of rice pigmentation and the nutritional quality of pigmented rice [4,18,19,20]. However, few comparative metabolomics studies have been conducted on these different colored rice varieties. Moreover, gene–metabolite network analyses in rice are lacking, which limits the efforts of breeders to develop novel rice varieties with desirable nutritional and medicinal values and improve active compounds for rice value addition [21]. Therefore, a comprehensive characterization of the metabolic profile differences between different colored rice grains is of great interest. It will reveal the spatial metabolite profile differences for the science-based use of different colored rice. It will also generate valuable data to investigate gene–metabolite interactions that may be useful for quality breeding purposes.

Widely targeted metabolite profiling is an advanced omics tool used to explore the metabolome of plant-derived products, resulting in an accurate qualitative and quantitative identification of a wide range of metabolites and a thorough understanding of phenotypic diversity in plants [22,23,24,25]. It has previously been applied to rice to elucidate the variation in metabolite profiles associated with phenotypic changes [24,26]. However, the study by Zhang et al. did not provide insights into the distribution and variation of nutraceuticals [26]. Moreover, only black and red rice grains were analyzed and compared with glutinous rice. Therefore, it would be more appropriate to include other colored rice and analyze non-glutinous rice separately.

The present study applied UHPLC–MS/MS (ultra-high performance liquid chromatography–mass spectroscopy)-based widely targeted metabolomics to comprehensively investigate variations in the metabolite profiles of long grain/non-glutinous black, red, green, and white rice seeds. Our objectives were to identify all DAMs (differentially accumulated metabolites) and major differentially regulated pathways among the four different colored rice varieties. In addition, we aimed to unveil the variational characteristics of major bioactive compounds and characteristic secondary metabolites in green rice. Our findings provide fundamental data that may allow the use of different colored rice in specific dietary programs.

## 2. Materials and Methods

### 2.1. Plant Materials and Reagents

**Figure 1:**
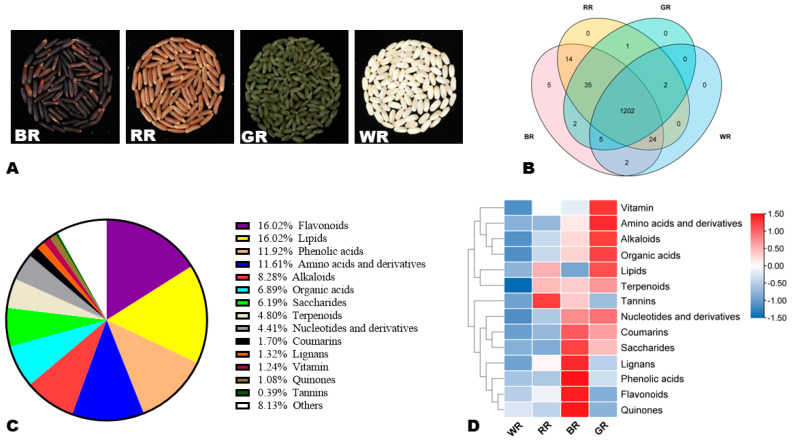
Morphology and variation of metabolites in the four different colored rice grains. (**A**) Images of the black, red, green, and white rice grains analyzed. (**B**) Venn diagram indicating the numbers of common and specific metabolites in the four rice varieties. (**C**) Classification of the 1292 identified metabolites. (**D**) Accumulation patterns of metabolite classes in the different colored rice grains. BR, black rice; RR, red rice; GR, green rice; and WR, white rice.

Four different colored rice varieties, including Haifeng Heidao No.3 (black, BR), Hainonghong No.2 (red, RR), Boyou 225 (white, WR), and LM8 (green, GR), were analyzed in this study (Figure 1A). All four rice varieties are long-grain and non-glutinous. BR, RR, and WR are new elite commercial indica rice germplasm resources in Hainan province. They were provided by the Hainan Academy of Agricultural Sciences (Hainan, China). GR was sourced from the International Rice Research Institute and gifted by the Chinese Academy of Agricultural Sciences (Beijing, China). The four varieties were carefully selected based on preliminary investigations to represent the different colored rice. All rice varieties were cultivated in Hainan (China) during the same season and under the same conditions. Thirty days after flowering, the grains were harvested and collected in triplicate for each variety. Each replicate was a mixture of grains from eight individual plants. Samples were dried in the sun to 10–11% water content and then stored in the dark at −80 °C until used. Prior to the UHPLC–MS analysis, the hulled grains were dehulled manually to obtain brown rice grains. Any broken grain was removed. All chemicals were purchased from Merck Company (Darmstadt, Germany), while metabolite standards were from Sigma-Aldrich (St. Louis, MO, USA) or BioBioPha (Kunming, China).

### 2.2. Metabolite Extraction and UHPLC–MS/MS Analysis

Seed samples were freeze-dried by a vacuum freeze-dryer (Scienta-100F) and subsequently reduced to flour with a mixer mill (MM 400, Retsch, Haan, Germany). The crushing was operated at 30 Hz for 1.5 min. Then, 100 mg of each sample’s flour was extracted for 12 h (constant shaking in darkness) at 4 °C with 1.2 mL methanol 70%, followed by centrifugation (15 min at 15,000× *g*) and collection of supernatants separately. The extracts were filtrated (0.22 μm micropore membrane, SCAA-104, ANPEL, Shanghai, China) and stored at −20 °C up to the UHPLC-ESI -MS/MS-based widely targeted metabolomics analysis at MWDB (Metware Biotechnology Co., Ltd., Wuhan, China) [24,25,26]. QC (quality control) samples were generated by mixing equal volumes of all extracts. A QC sample was analyzed every six samples to assess the repeatability of the measurement process. 

Metabolite profiling was conducted according to previously described methods [25,26]. The detailed information on the liquid phase and MS conditions is provided in Table S1. The m/z range for metabolite detection was 50–1250 Da.

### 2.3. Identification and Quantification of Metabolites

The metabolites were qualitatively identified based on the spectrum information, retention times relative to external standards, and mass spectra. We compared the accurate isolate precursor ions (Q1), fragment ions (Q3), retention times, and fragmentation patterns with standards to analyze the primary and secondary MS data (Sigma-Aldrich, St. Louis, MO, USA). Finally, all identified metabolites were confirmed by searching in the MWDB in-house database and public databases (KNApSAcK, MoTo DB, MassBank, METLIN, and HMDB) [24,26]. 

Metabolite quantification was conducted using the multiple reaction monitoring (MRM) mode, which consisted of triple quadrupole (QqQ) mass spectrometry analysis. In the MRM mode, the quadrupole first searched for the parent ions of target substances while screening any ions derived from substances of different molecular weights to dismiss their interference. Further, the precursor ions were fragmented to form many fragment ions. The fragment ions were then filtered through QqQ to eliminate interference from non-target ions and precisely select single-fragment ions with the desired characteristics. Next, all the obtained mass spectrum peaks were subjected to area integration. Using MultiaQuantTM (AB Sciex™, Framingham, MA, USA) software, we integrated and corrected the mass spectra peaks of the same metabolite in different samples. The area of each peak represents the relative content of the corresponding substance. Finally, all the integration data of the peak area were exported and saved.

### 2.4. Data Analysis

We first assessed the quality of the data, and substances with large deviations (CV value greater than 0.5) were eliminated. Further, the Zscore was used to standardize the data. Thereafter, we conducted multivariate analyses, including PCA (principal component analysis), HCA (hierarchical clustering analysis), OPLS-DA (orthogonal partial least squares discriminant analysis), and correlations analyses in R (version 4.3.0) using the packages prcomp, pheatmap, MetaboAnalystR, and cor, respectively. Significant DAMs were revealed using the R-program ggplot2. The filtering thresholds were Log_2_FC ˃ 1, VIP ≥ 1, and *p*-value < 0.05. VIP (variable important in projection) values were sourced from the OPLS-DA. Functional annotation of DAMs was performed via KEGG (Kyoto Encyclopedia of Genes and Genomes) analysis (http://www.kegg.jp/kegg/pathway.html). Significantly enriched pathways were detected by metabolite set enrichment analysis and the hypergeometric test. GraphPad Prism (v9.0., La Jolla, CA, USA) and Microsoft Excel 2021 were used for data processing and the construction of graphs. TBtools (v1.09867) was used to construct Venn diagrams and heatmaps [27]. 

## 3. Results

### 3.1. Metabolic Profiles of Black, Red, Green, and White Rice Grains

To unveil the distribution and variation of metabolites in rice grains of different colors, we carried out widely targeted metabolic profiling of four rice types, including BR, RR, GR, and WR (Figure 1A). The total ion chromatograms of some identified compounds in the quality control (QC) samples are shown in Figure S1. The repeatability of the experiment was confirmed by the high correlations (r ≥ 0.98) recorded between QC samples (Figure S2A). We identified a total of 1292, including 1202 common metabolites in BR, RR, GR, and WR (Figure 1B, Table S2). Five flavonoid compounds, including quercetin-3-*O*-(6″-*O*-acetyl)glucoside, 2′-hydoxy-5-methoxygenistein-4′,7-*O*-diglucoside, peonidin-3-*O*-(6″-*O*-acetyl)glucoside, cyanidin-3-*O*-(6″-*O*-acetyl)glucoside, and bracteatin, were specific to black rice (Figure 1B). 

In order to explore the global rice grain metabolome, we proceeded with the classification of metabolites. The results showed that the rice grain metabolome is mainly dominated by flavonoids (16.02%), lipids (16.02%), phenolic acids (11.92%), amino acids and derivatives (11.61%), alkaloids (8.28%), organic acids (6.89%), and saccharides (6.19%) (Figure 1C). Vitamins, lignans, and tannins accounted for 1.24%, 1.32%, and 0.39%, respectively (Figure 1C). Further, we computed the sum of the relative contents of all metabolites within each category and examined their variation between the four rice types (Figure 1D). We found that GR had the highest relative content of vitamins, amino acids and derivatives, organic acids, alkaloids, lipids, and nucleotides and derivatives (Figure 1D). Meanwhile, BR had the highest relative content of coumarins, saccharides, lignans, phenolic acids, flavonoids, and quinones (Figure 1D). RR exhibited the highest content of tannins (Figure 1D).

### 3.2. Variability of Metabolites in the Different Colored Rice Grains

**Figure 2:**
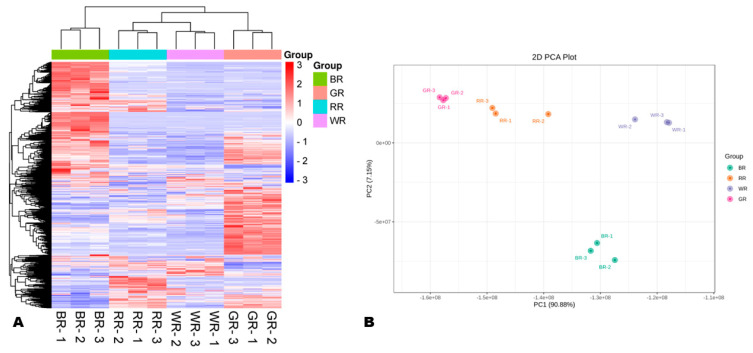
Multivariate analysis of the metabolite profiles. (**A**) Hierarchical clustering analysis (HCA). (**B**) Principal component analysis (PCA). BR, black rice; RR, red rice; GR, green rice; and WR, white rice.

To effectively reveal the variability of metabolites between the different colored rice grains, we conducted correlation analysis and a set of multivariate data analyses (Figure 2, Figures S2B and S3). As shown in Figure 2A, the HCA revealed remarkable differences between the metabolite profiles of BR, RR, GR, and WR. BR and GR exhibited the highest relative content of many metabolites compared to RR and WR (Figure 2A). Notably, BR samples were clustered separately, indicating that their metabolite profile is entirely different compared to other colored rice grains (Figure 2A). The PCA also showed that the metabolite profiles of BR, RR, GR, and WR were different (Figure 2B). GR and WR samples gathered closely on the PCA plot and showed high correlations, indicating some extend of similarity between their metabolite profiles (Figure 2B and Figure S2B). The OPLS-DA analysis results indicated that the R^2^Y and Q^2^ of all pairwise comparisons were equal or close to one, confirming the observed metabolite variability trends between the four different colored rice types (Figure S3A–F).

### 3.3. Differentially Accumulated Metabolites and KEGG Enrichment

**Figure 3:**
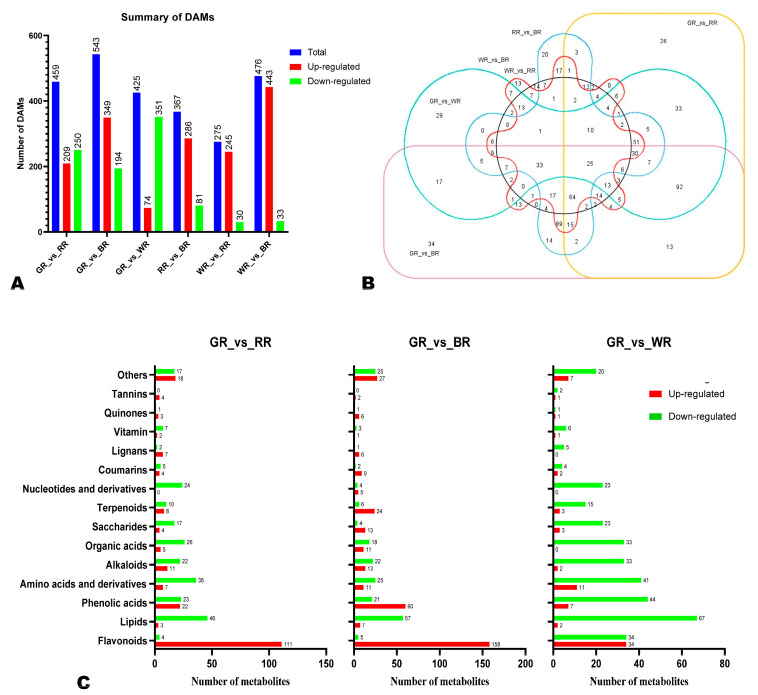
Differentially accumulated metabolites (DAMs). (**A**) Summary of the numbers of DAMs. (**B**) Venn diagram exhibiting the number of the key DAMs across all pairwise comparisons. (**C**) Classification of the DAMs. Up-regulation for “X.vs.Y” indicates that the metabolite has higher relative content in Y. BR, black rice; RR, red rice; GR, green rice; and WR, white rice.

To identify the metabolites that were differentially accumulated between BR, RR, GR, and WR, we extracted the VIP values from the OPLS-DA and filtered out all DAMs in pairwise comparisons by using the thresholds of VIP ≥ 1, Log_2_FC > 1, and *p*-value < 0.05. Our analysis revealed a total of 543 (349 up-regulated in BR), 459 (209 up-regulated in RR), 425 (74 up-regulated in WR), 476 (443 up-regulated in BR), 275 (245 up-regulated in RR), and 467 (386 up-regulated in BR) DAMs in pairwise comparison between GR_vs_BR, GR_vs_RR, GR_vs_WR, WR_vs_BR, WR_vs_RR, and RR_vs_BR, respectively (Figure 3A and Figure S4). We then constructed a Venn diagram to uncover key overlapped DAMs that could serve as potential biomarkers to differentiate between rice grains of different colors (Figure 3B). In total, twenty-five overlapped DAMs, including seventeen flavonoids, four alkaloids, one phenolic acid, and two terpenoids, were identified between all pairwise comparisons (Figure 3B and Table 1). Meanwhile, we detected 99 overlapped DAMs in pairwise comparisons between BR, RR, and GR against WR (Figure S5 and Table S3). The classification of identified DAMs between all pairwise comparisons showed that flavonoids, lipids, and phenolic acids were the most significantly differentially accumulated (Figure 3C and Figure S6). Many lipids, amino acids and derivatives, organic acids, and alkaloids were up-regulated in GR compared to other colored rice grains (Figure 3C and Figure S6). Table 1 provides a list of the 25 key differentially accumulated metabolites that could serve as potential discriminatory biomarkers of different colored rice grains.

**Figure 4:**
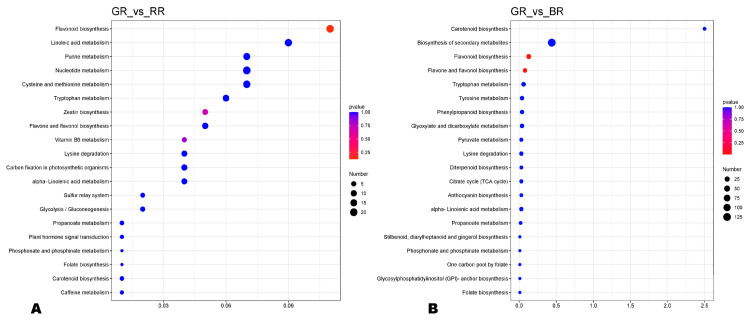
KEGG annotation and enrichment results of DAMs in pairwise comparisons between (**A**) GR_vs_RR and (**B**) GR_vs_BR. BR, black rice; RR, red rice; GR, green rice; and WR, white rice.

To uncover the major differentially regulated pathways (DRPs) between BR, RR, GR, and WR, we performed KEEG analysis of DAMs (Figure 4 and Figure S7). The main DRPs between GR and RR were flavonoid biosynthesis, zeatin biosynthesis, and vitamin B6 metabolism (Figure 4A). Meanwhile, flavonoid biosynthesis and flavone and flavonol biosynthesis were the most common DRPs between GR and BR (Figure 4B). As shown in Figure S7A, the main DRPs between GR and WR were zeatin biosynthesis and linoleic acid metabolism. Flavonoid (flavone and flavonol) biosynthesis was the most enriched pathway between WR and BR (Figure S7B). The most common DRPs between WR and RR were flavonoid biosynthesis, flavone and flavonol biosynthesis, and anthocyanin biosynthesis (Figure S7C). The main enriched metabolic pathways between RR and BR were biosynthesis of secondary metabolites, flavonoid (flavone, flavonol, and anthocyanin) biosynthesis, and purine metabolism (Figure S7D).

### 3.4. Variation Characteristics of Major Bioactive Compounds in Black, Red, Green, and White Rice Grains

**Figure 5:**
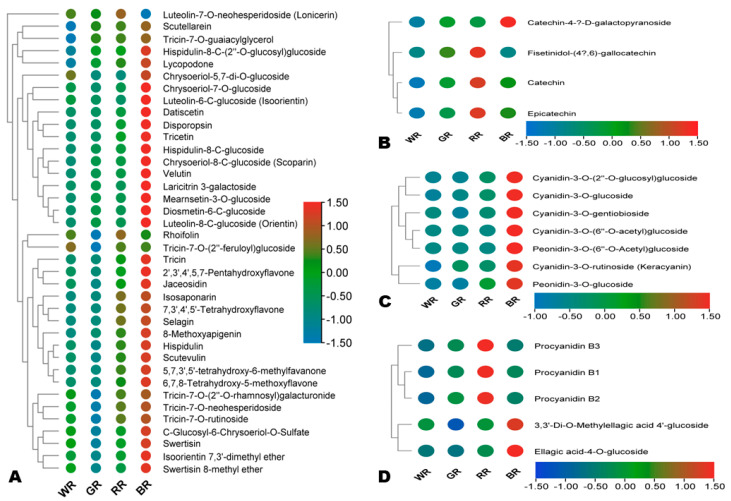
Variation of major differentially accumulated bioactive flavonoids and tannins in the four different colored rice grains. (**A**) Flavones; (**B**) flavanols; (**C**) anthocyanidins; and (**D**) procyanidins and tannins. BR, black rice; RR, red rice; GR, green rice; and WR, white rice.

**Figure 6:**
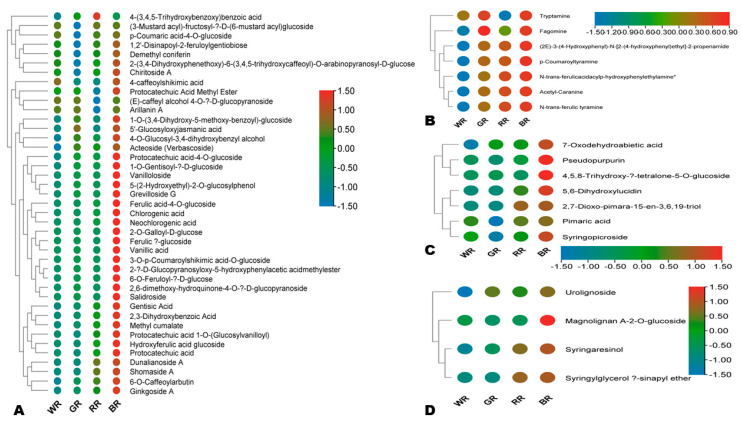
Variation in major differentially accumulated bioactive phenolic acids (**A**), alkaloids (**B**), quinones and terpenoids (**C**), and lignans (**D**) in the four different colored rice grains. BR, black rice; RR, red rice; GR, green rice; and WR, white rice.

To reveal the variation of bioactive compounds among the different colored rice grains, we filtered out major differentially accumulated (MDA, |Log_2_FC| ≥ 5) flavonoids, alkaloids, phenolic acids, lignans, terpenoids, coumarins, and quinones. Amino acids were not included because they exhibited very low fold changes. In total, 182 active compounds were screened out and examined (Figure 5, Figure 6 and Figure S8). Almost all the MDA flavones, flavanones, flavonols, anthocyanins, flavanonols, isoflavones, and chalcones had the highest relative content in BR, followed by RR (Figure 5A,C and Figure S8A–E). The MDA anthocyanins were glycosylated cyanidins and peonidins (Figure 5C). All MDA procyanidins (procyanidin B1, B2, and B3) and three MDA flavanols, including catechin, epicatechin, and fisetinidol-(4,6)-gallocatechin, had the highest relative content in RR (Figure 5B,D). 3,3′-di-*O*-methylellagic acid 4′-glucoside and ellagic acid-4-*O*-glucoside (tannins) had the highest relative content in BR, followed by RR and WR (Figure 5D).

Similarly, all the MDA phenolic acids, alkaloids, quinones, terpenoids, lignans, and coumarins had the highest relative content in BR, except for 4-(3,4,5-trihydroxybenzoxy) benzoic acid (phenolic acid) and fagomine (alkaloid), which exhibited the highest relative content in RR and GR, respectively (Figure 6A–D and Figure S9). RR and GR exhibited higher relative contents of MDA alkaloids, including (2E)-3-(4-hydroxyphenyl)-N-[2-(4-hydroxyphenyl)ethyl]-2-propenamide, acetyl-caranine, N-trans-ferulic tyramine, N-trans-ferulicacidacylp-hydroxyphenylethylamine, *p*-coumaroyltyramine, and fagomine, than WR (Figure 6B). The MDA phenolic acids and coumarins exhibited almost similar patterns in WR, GR, and RR (Figure 6A and Figure S9). Compared to WR and GR, RR showed higher relative contents of syringaresinol and syringylglycerol β-sinapyl ether (lignans) (Figure 6D).

### 3.5. Distribution and Variation of Vitamins in the Different Colored Rice Grains

**Figure 7:**
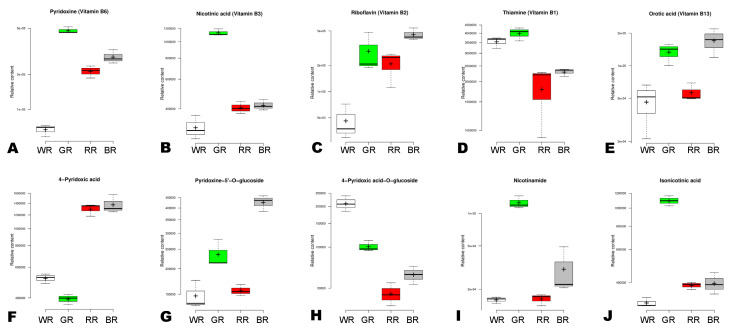
(**A**–**J**) Variation of ten differentially accumulated vitamins in the four different colored rice grains. BR, black rice; RR, red rice; GR, green rice; and WR, white rice.

Vitamins are one of the most important bioactive compounds in rice. To reveal the variation characteristics of vitamins in BR, RR, GR, and WR, we examined the relative contents of the differentially accumulated vitamins. Vitamin B6, vitamin B3, vitamin B1, nicotinamide, and isonicotinic acid had the highest levels in GR (Figure 7A,B,D,I,J). Meanwhile, vitamin B2, vitamin B13, and pyridoxine-5′-*O*-glucoside had the highest contents in BR, followed by GR (Figure 7C,E,G). 4-pyridoxic acid exhibited the highest content in BR, followed by RR and WR (Figure 7F). 4-pyridoxic acid-*O*-glucoside had the highest relative content in WR, followed by GR, BR, and RR (Figure 7H). To explore the potential impact of pigment synthesis on variation in vitamin content, we investigated the correlation between vitamins, anthocyanidins, and proanthocyanidins (Figure S10). The results showed no significant correlations between the three classes of metabolites (Figure S10A). Among the vitamins, thiamine (vitamin B1, pmb0952) and 4-pyridoxic acid (pme2596) showed a significant negative correlation (Figure S10A,B).

### 3.6. Key Secondary Metabolites in Green Rice

**Figure 8:**
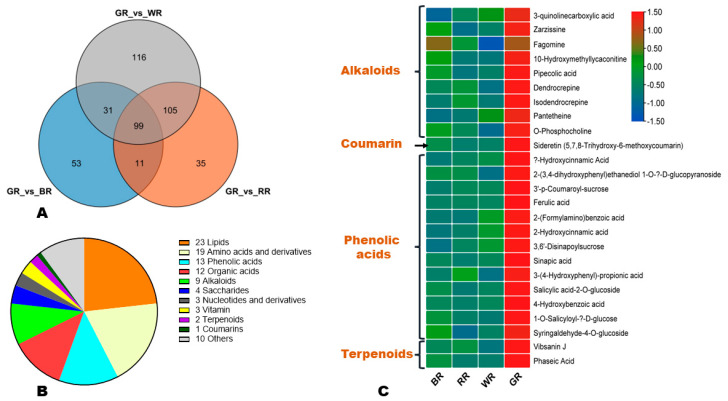
Key secondary metabolites in green rice. (**A**) Venn diagram indicating the 99 DAMs highly accumulated in GR than other colored rice grains. (**B**) Classification of the 99 key up-regulated DAMs in GR. (**C**) Heatmap of the variation of key 25 GR’s secondary metabolites in the four different colored rice grains. BR, black rice; RR, red rice; GR, green rice; and WR, white rice.

To unveil key secondary metabolites in green rice, we constructed a Venn diagram among up-regulated DAMs in GR (Figure 8A). The result revealed that 99 DAMs, including 25 secondary metabolites, 23 lipids, 19 amino acids, 12 organic acids, and 4 saccharides, were specifically highly accumulated in GR compared to other colored rice grains (Figure 8A,B). The twenty-five secondary metabolites include nine alkaloids (3-quinolinecarboxylic acid, zarzissine, fagomine, 10-hydroxymethyllycaconitine, pipecolic acid, dendrocrepine, isodendrocrepine, pantetheine, and *O*-phosphocholine), thirteen phenolic acids (α-hydroxycinnamic acid, 3′-*p*-coumaroyl-sucrose, ferulic acid, 2-hydroxycinnamic acid, sinapic acid, 3-(4-hydroxyphenyl)-propionic acid, salicylic acid-2-O-glucoside, 4-hydroxybenzoic acid, etc.), one coumarin (sideretin), and two terpenoids (vibsanin J and phaseic acid) (Figure 8C). Fagomine is part of the key DAMs and could be used as a biomarker for GR. 

## 4. Discussion

Value addition in rice is of global interest, as it will significantly contribute to food security and reduce the prevalence of lifestyle diseases. Therefore, it is vital to identify all the active phytochemicals in rice and understand their diversity, distribution, and variation within different rice varieties. Although some studies have been conducted to elucidate the phytochemical profiles of different colored rice grains [5,6,7,28,29,30], a comparative analysis of the global metabolome underlying phenotypic and bioactivity variations is lacking, limiting the science-based exploitation of the potential of rice strains. Thus, this study applied widely targeted metabolomics to comprehensively reveal the metabolic profile differences between BR, RR, GR, and WR, with a particular focus on the variation characteristics of major nutraceuticals and vitamins and key secondary metabolites in GR. 

Compared to Zhang et al., who identified 732 metabolites in red, black, glutinous, and typical white rice [26], we identified and chemically characterized 1292 metabolites in this study, providing a more complete exposure to rice phytochemical composition. In addition, this study includes a green rice variety, which provides the opportunity for future gene discovery and rice quality improvement. Metabolites, as well as metabolite classes, exhibited different accumulation patterns in the four rice grains of different colors. Supportively, pairwise OPLS-DA analysis revealed that the metabolite profiles of BR, RR, GR, and WR were very different from each other. We identified 275–543 DAMs, the majority of which were up-regulated in BR, followed by RR, WR, and GR. These results present evidence of differential regulation of metabolic processes in different colored rice varieties. Moreover, they indicate a great opportunity for candidate gene mining and targeted improvement of rice, which contains active compounds. 

KEGG analysis of the DAMs disclosed that they were primarily enriched in flavonoid (flavone, flavonol, and anthocyanin) biosynthesis, biosynthesis of cofactors, biosynthesis of secondary metabolites, zeatin biosynthesis, and vitamin B6 metabolism, inferring that these pathways might be the major differentially regulated pathways in the different colored rice. Previous studies have shown that the flavonoid pathway is the most differently regulated among different colored rice, and some candidate genes have been identified [4,18,26,31,32]. Notably, the regulation of anthocyanin biosynthesis in BR has been the subject of many studies [18,19,20]. Further comparative analysis of the dynamic expression of genes related to these pathways at the transcriptional levels between rice of different colors is required for an in-depth understanding of variation in rice quality and the complete dissection of the underlying molecular regulatory networks. We also identified 25 key DAMs that could serve as potential metabolic biomarkers to differentiate between rice of different colors. They may also help authenticate and assess the quality of rice-derived products [33]. Furthermore, these key DAMs may represent references for analyzing the gene–metabolite networks that govern variation in rice quality.

Pigmented rice, especially BR varieties, contain higher levels of flavonoids, anthocyanins, and phenolic acids and possess diverse pharmacological attributes, e.g., anti-diabetic, anti-hyperlipidemia, antioxidant, anticancer, cardioprotective, anti-osteosclerosis, antiallergic, etc., [6,7,9,11,12,14,34]. Herein, we found that over 170 MDA metabolites, including flavones, flavanones, flavonols, isoflavones, anthocyanidins, chalcones, phenolic acids, alkaloids, quinones, lignans, coumarins, and terpenoids, have higher contents in BR, followed by RR. Consistent with the report by Periera-Caro et al., we found that procyanidins (B1, B2, and B3) had the highest contents in RR [16]. These results confirm that BR and RR are excellent materials to integrate into food pharmacy formulations to treat or prevent chronic diseases. Moreover, they show that BR and RR may represent great resources for producing anthocyanin- and proanthocyanin-derived medicines. Anthocyanins and proanthocyanins are natural polyphenols with recorded health benefits, e.g., anti-hyperlipidemia, anti-diabetic, cardioprotective, anticancer, etc. [10,35,36,37]. GR exhibited the highest relative content of vitamins (B6, B3, B1, nicotinamide, and isonicotinic acid), lipids, amino acids and derivatives, alkaloids, and organic acids, indicating it may be an excellent source of essential nutrients and essential oils. It may also represent a health-promoting material due to its high alkaloid content, terpenoid and phenolic acid profiles, and key 25 secondary metabolites. Alkaloids and terpenoids are important nutraceuticals for preventing cancers and neurological diseases [38,39,40]. The key secondary metabolites in GR may also serve to authenticate green rice-derived products from other colored rice grains. For example, fagomine has been identified as a key secondary metabolic marker for discriminating Wuchang Daohuaxiang rice among Chinese rice [41]. More studies involving green rice varieties are required to identify novel key genetic resources for an integrated improvement of rice quality values.

## 5. Conclusions

In summary, this study provides an in-depth understanding of metabolite variation in black, red, green, and white rice grains. It revealed the accumulation patterns of major nutraceuticals and the distribution characteristics of vitamins in the four different colored rice varieties, and identified 25 potential discriminatory biomarkers. The most differentially regulated pathway includes flavonoid (flavone, flavonol, and anthocyanin) biosynthesis and the biosynthesis of cofactors. Black rice showed an excellent profile of bioactive compounds and is confirmed to be an essential ingredient for therapeutic purposes. Red rice can be recommended as a rich source of procyanidins, catechin, and their derivatives. Meanwhile, green rice can be an excellent resource of vitamins, alkaloids, and amino acids. In addition, we identified twenty-five key secondary metabolites in green rice. Our results offer fundamental resources for deciphering gene–metabolite interactions that drive variation in rice quality traits. Moreover, they provide important data to promote the science-based use of different colored rice in diverse industries.

## Figures and Tables

**Table 1:** List of the 25 key differentially accumulated metabolites that could serve as potential discriminatory biomarkers of different colored rice grains.

Compounds	Class	KEGG ID	Log_2_FC					
			GR_vs_BR	GR_vs_RR	GR_vs_WR	RR_vs_BR	WR_vs_BR	WR_vs_RR
*p*-Coumaroyltyramine	Alkaloids	-	3.06	1.10	−11.95	1.96	15.01	13.05
N-Feruloylserotonin	Alkaloids	-	1.16	−1.89	−3.66	3.05	4.82	1.77
Fagomine	Alkaloids	C10144	−1.18	−7.21	−15.92	6.03	14.74	8.71
(2E)-3-(4-Hydroxyphenyl)-N-[2-(4-hydroxyphenyl)ethyl]-2-propenamide	Alkaloids	-	3.01	1.07	−11.43	1.94	14.44	12.50
6,7-dihydroxy-1,3-dimethoxyxanthen-9-one	Flavonoids	-	9.73	3.26	−1.50	6.47	11.23	4.76
1,8-dihydroxy-4,5-dimethoxy-3-{[(2s,3r,4s,5s,6r)-3,4,5-trihydroxy-6-(hydroxymethyl)oxan-2-yl]oxy}xanthen-9-one	Flavonoids	-	14.62	11.16	7.78	3.46	6.84	3.38
Tamarixin	Flavonoids	-	16.06	8.73	10.03	7.33	6.04	−1.30
Eriodictyol	Flavonoids	C05631	9.43	2.85	−7.35	6.58	16.78	10.20
2′,3′,4′,5,7-Pentahydroxyflavone	Flavonoids	-	11.84	3.99	1.24	7.85	10.60	2.75
Hesperetin	Flavonoids	C01709	9.28	2.32	−7.13	6.96	16.41	9.45
Tricin-7-*O*-rutinoside	Flavonoids	-	7.23	5.59	3.74	1.63	3.48	1.85
Isorhamnetin	Flavonoids	C10084	9.91	1.75	−1.68	8.16	11.59	3.43
Homoeriodictyol	Flavonoids	C09756	9.40	1.98	−7.66	7.42	17.06	9.64
2-Hydroxyxanthone	Flavonoids	-	4.98	2.07	−8.66	2.91	13.64	10.73
Homeriodictyol	Flavonoids	-	9.40	2.02	−8.08	7.37	17.48	10.11
Tamarixetin-3-*O*-rutinoside	Flavonoids	-	−11.02	1.64	−2.37	−12.66	−8.66	4.01
Tricin-7-*O*-neohesperidoside	Flavonoids	-	7.23	5.59	3.74	1.63	3.48	1.85
Pinobanksin	Flavonoids	C09826	4.20	2.55	−1.83	1.64	6.02	4.38
Jaceosidin	Flavonoids	-	8.74	3.89	1.47	4.85	7.27	2.42
Naringenin	Flavonoids	C00509	4.91	3.01	−1.33	1.91	6.24	4.34
2-Isopropylmalic acid	Organic acids	C02504	4.01	1.85	−7.47	2.16	11.48	9.32
Capillarisin	Others	C08999	10.15	2.06	−7.44	8.09	17.60	9.51
4-*O*-Glucosyl-3,4-dihydroxybenzyl alcohol	Phenolic acids	-	1.92	−1.55	−3.37	3.48	5.29	1.81
Syringopicroside	Terpenoids	-	16.61	10.26	8.88	6.35	7.73	1.38
Pimaric acid	Terpenoids	C09159	16.55	14.96	13.89	1.58	2.66	1.07

Note: For X_vs_Y, negative and positive values indicate up-regulated in X and Y, respectively.

## Data Availability

The original contributions presented in the study are included in the article/Supplementary Materials, further inquiries can be directed to the corresponding authors.

## Supplementary Materials

The following supporting information can be downloaded at: https://www.mdpi.com/article/10.3390/foods13121899/s1, Figure S1: Multiple reaction monitoring (MRM) graphs of QC samples showing the total ion current (TIC) of some identified metabolites; Figure S2: Correlation analysis between (A) the twelve samples and (B) QC samples; Figure S3: Permutation plots of OPLS-DA results of pairwise comparisons; Figure S4: Volcano plots of the DAMs in pairwise comparisons; Figure S5: Venn diagram exhibiting the number of overlapped DAMs when comparing pigmented rice against WR; Figure S6: Classification of the DAMs in other pairwise comparisons; Figure S7: KEGG annotation and enrichment results of DAMs in pairwise comparisons between GR_vs_WR, WR_vs_BR, WR_vs_RR, and RR_vs_BR, respectively; Figure S8: Variation of other major differentially accumulated bioactive flavonoids; Figure S9: Variation of differentially accumulated bioactive coumarins; Figure S10: Correlations between vitamins and major proanthocyanidins and anthocyanidins; Table S1: LC and MS conditions; Table S2: List of the 1292 identified metabolites; Table S3: List of the 99 overlapped DAMs when comparing pigmented rice against white rice.
