# Supplementary material for: Comparative Metabolic Profiling of Different Colored Rice Grains Reveals the Distribution of Major Active Compounds and Key Secondary Metabolites in Green Rice
Source: Foods. 2024 Jun 17;13(12):1899. doi: 10.3390/foods13121899 (PMC11202634; doi:10.3390/foods13121899)
Supplement: Supplementary file 1 [file foods-13-01899-s001.zip › Supplementary Figures.pdf]

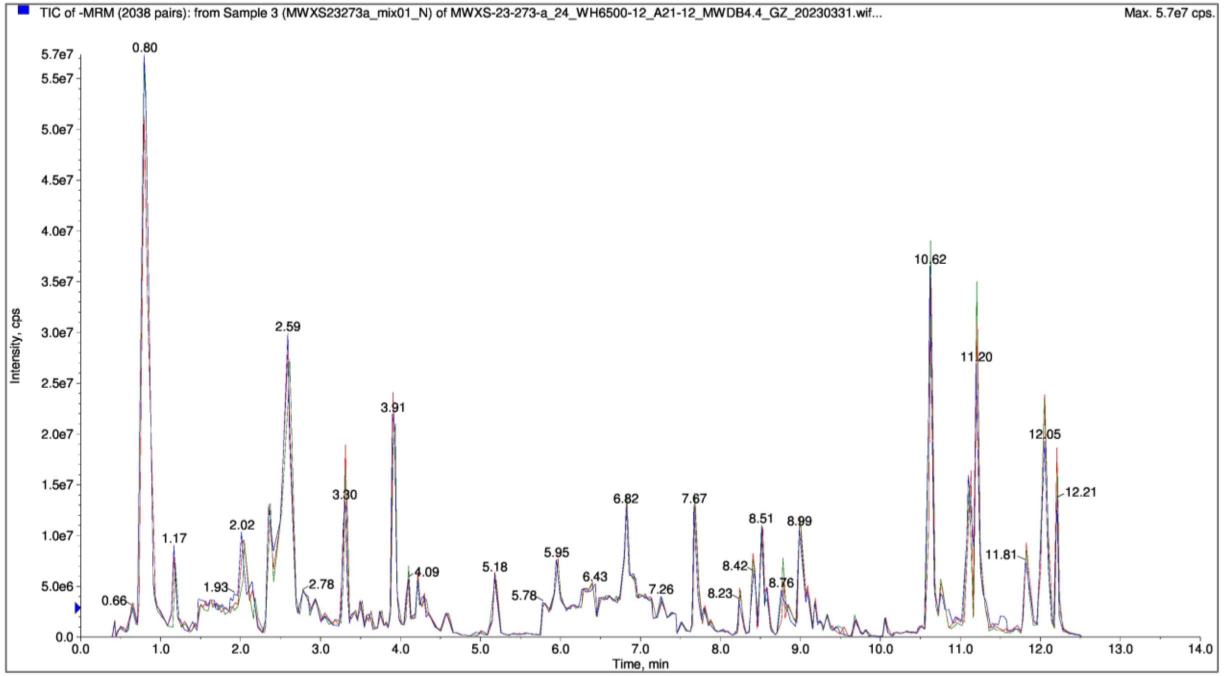

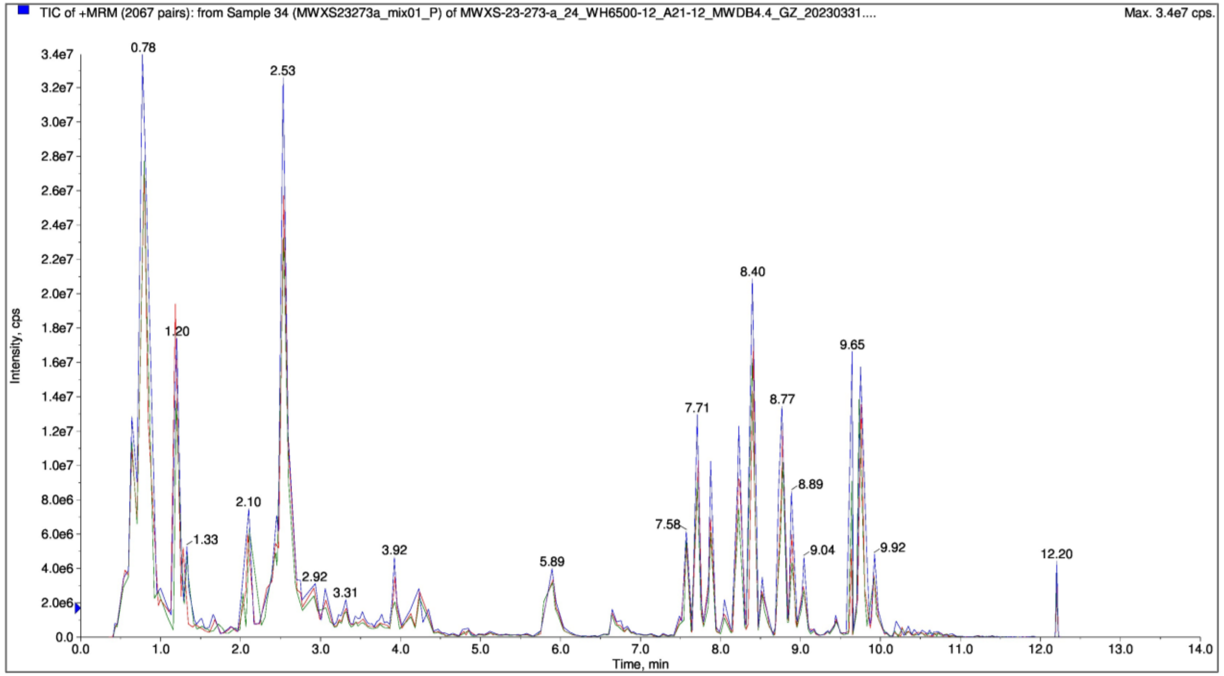

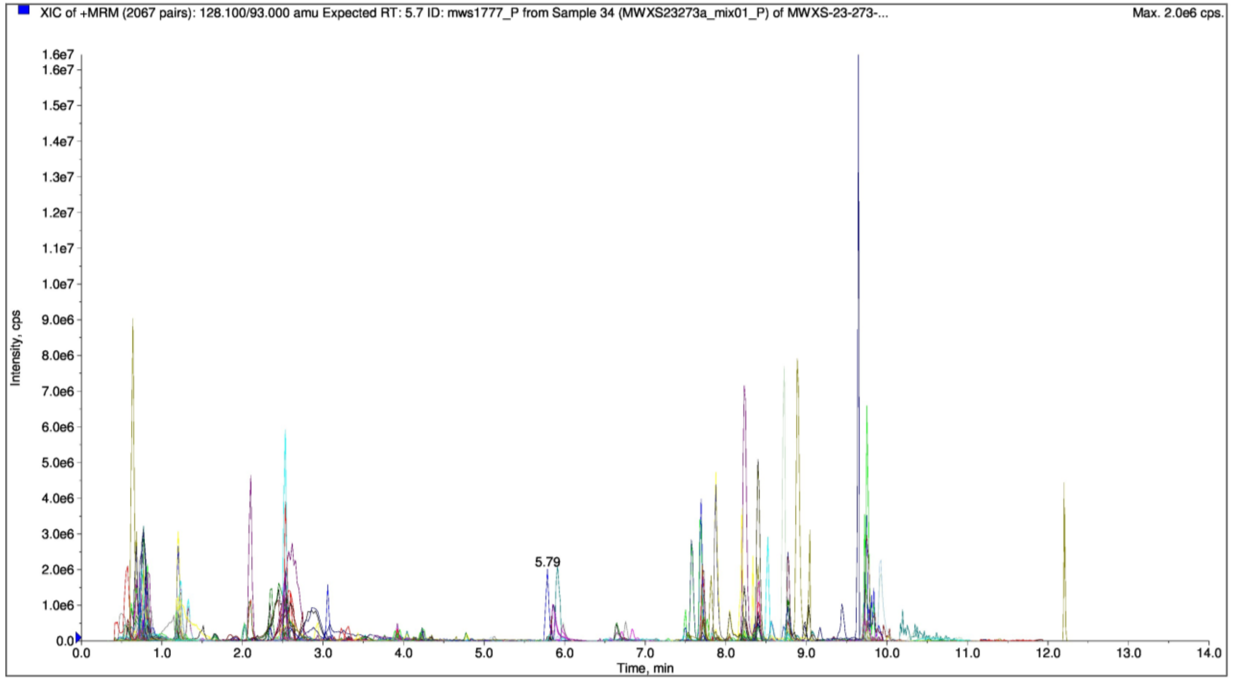

Fig S1. Multiple reaction monitoring (MRM) graphs of QC samples showing total Ions Current (TIC) of some identified metabolites. Each color represents the detected metabolites in the sample. The vertical axis of the graph represents the current ion intensity counts per second (cps), whereas the horizontal axis represents the retention time of metabolites per minute(min.).

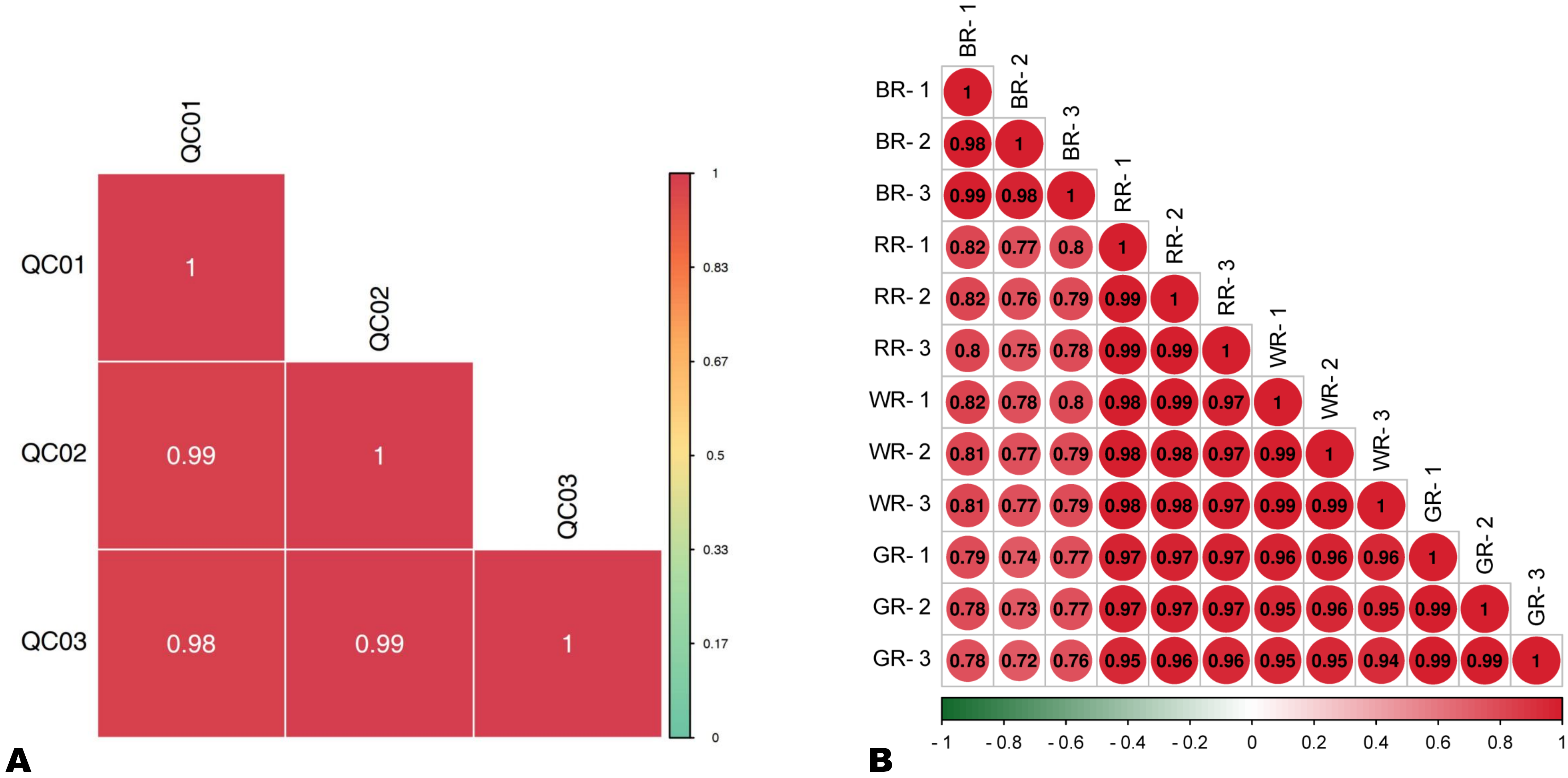

Fig S2. Correlations analysis between: (A) the twelve samples and (B) QC samples.
BR, black rice; RR, red rice; GR, green rice; WR, white rice.

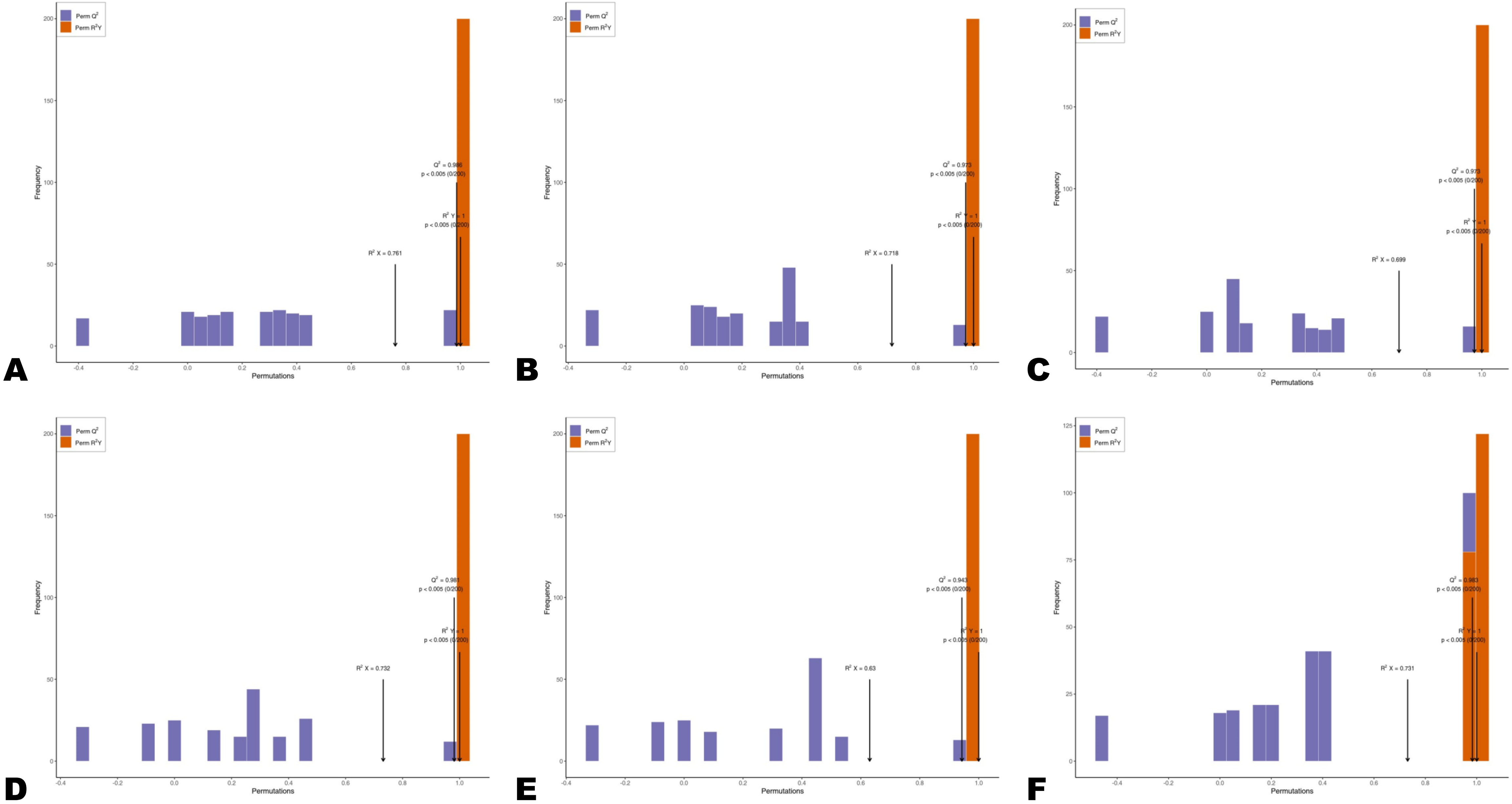

Fig. S3. (A)-(F) Permutation plots of OPLS-DA results of pairwise comparisons between GR_vs_BR, GR_vs_RR, GR_vs_WR, WR_vs_BR, WR_vs_RR, and RR_vs_BR, respectively. BR, black rice; RR, red rice; GR, green rice; WR, white rice.

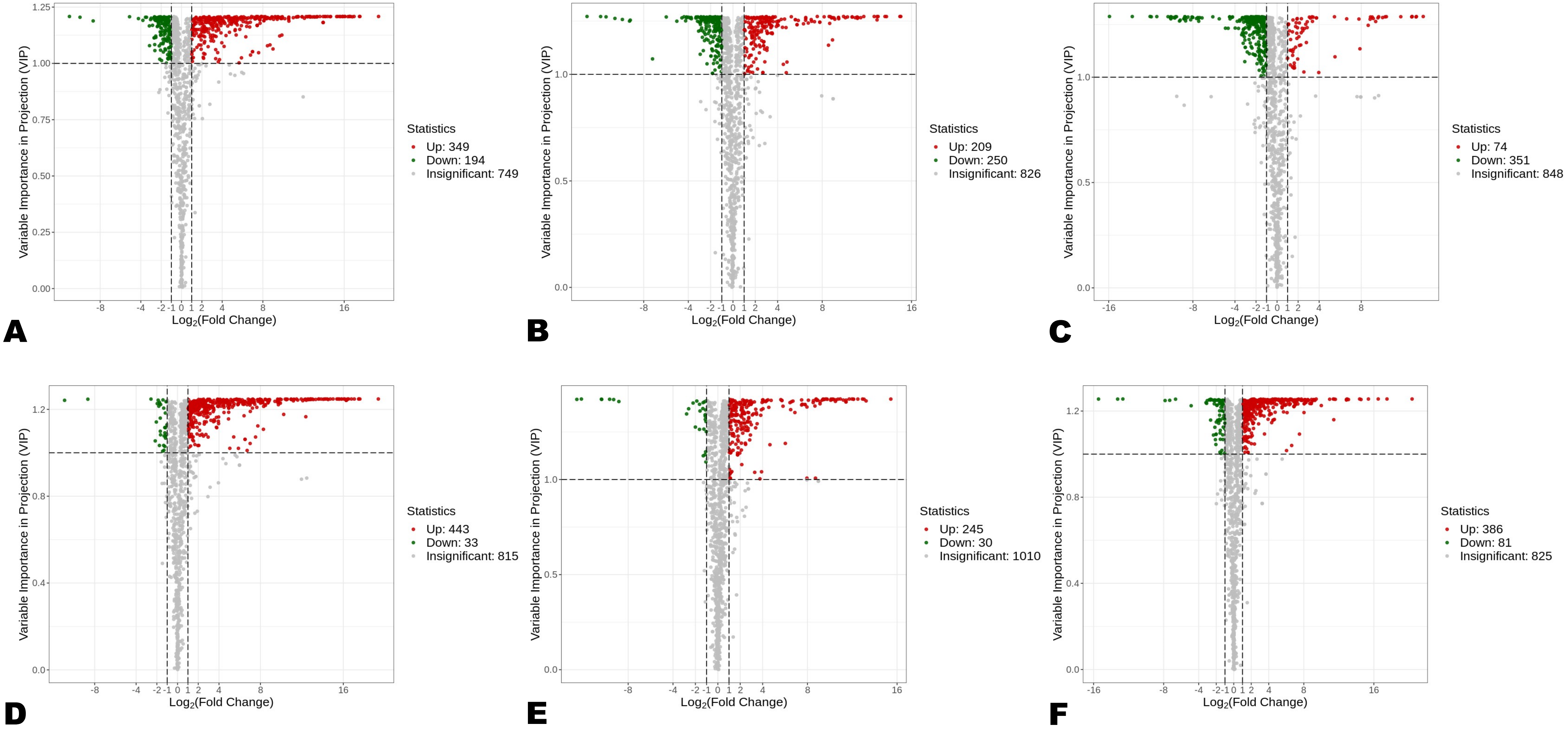

Fig. S4. Volcano plots of the DAMs in pairwise comparisons between GR_vs_BR, GR_vs_RR, GR_vs_WR, WR_vs_BR, WR_vs_RR, RR_vs_BR, respectively.

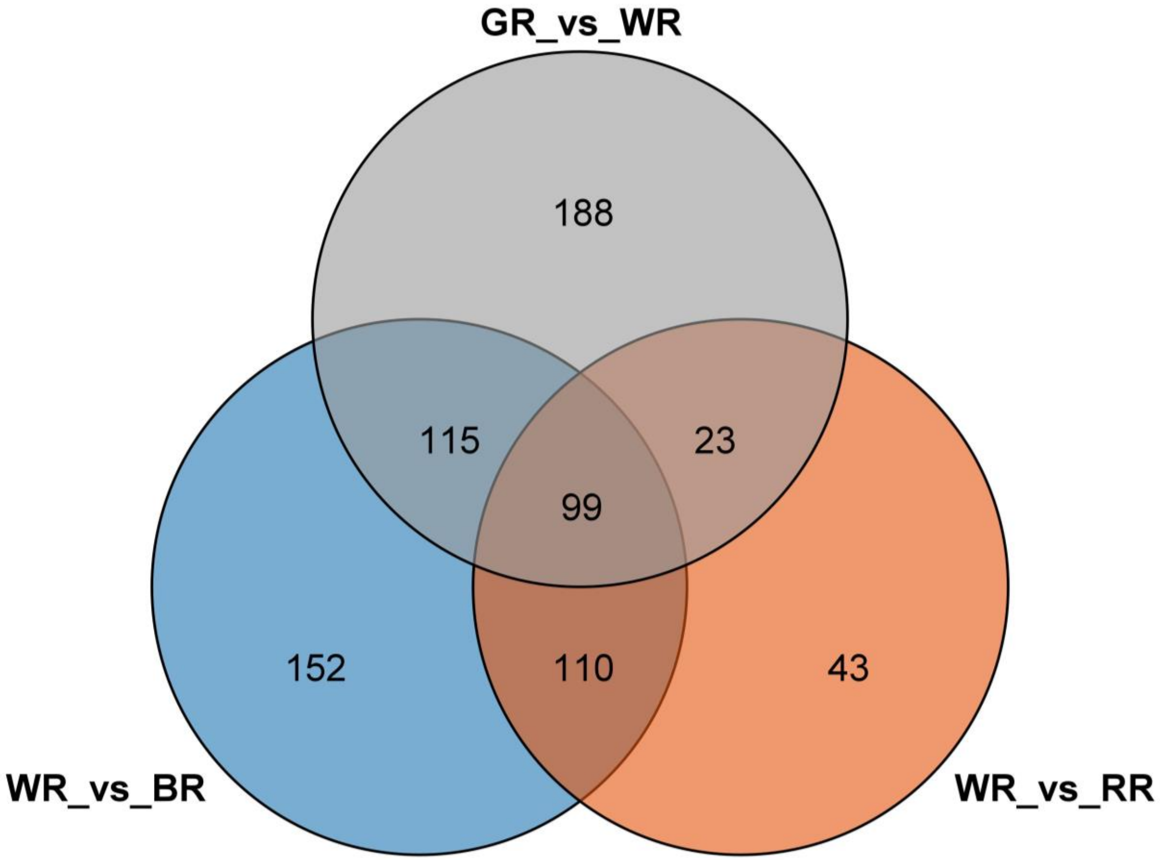

Fig. S5. Venn diagram exhibiting the number of overlapped DAMs when comparing pigmented rice against the WR. BR, black rice; RR, red rice; GR, green rice; WR, white rice.

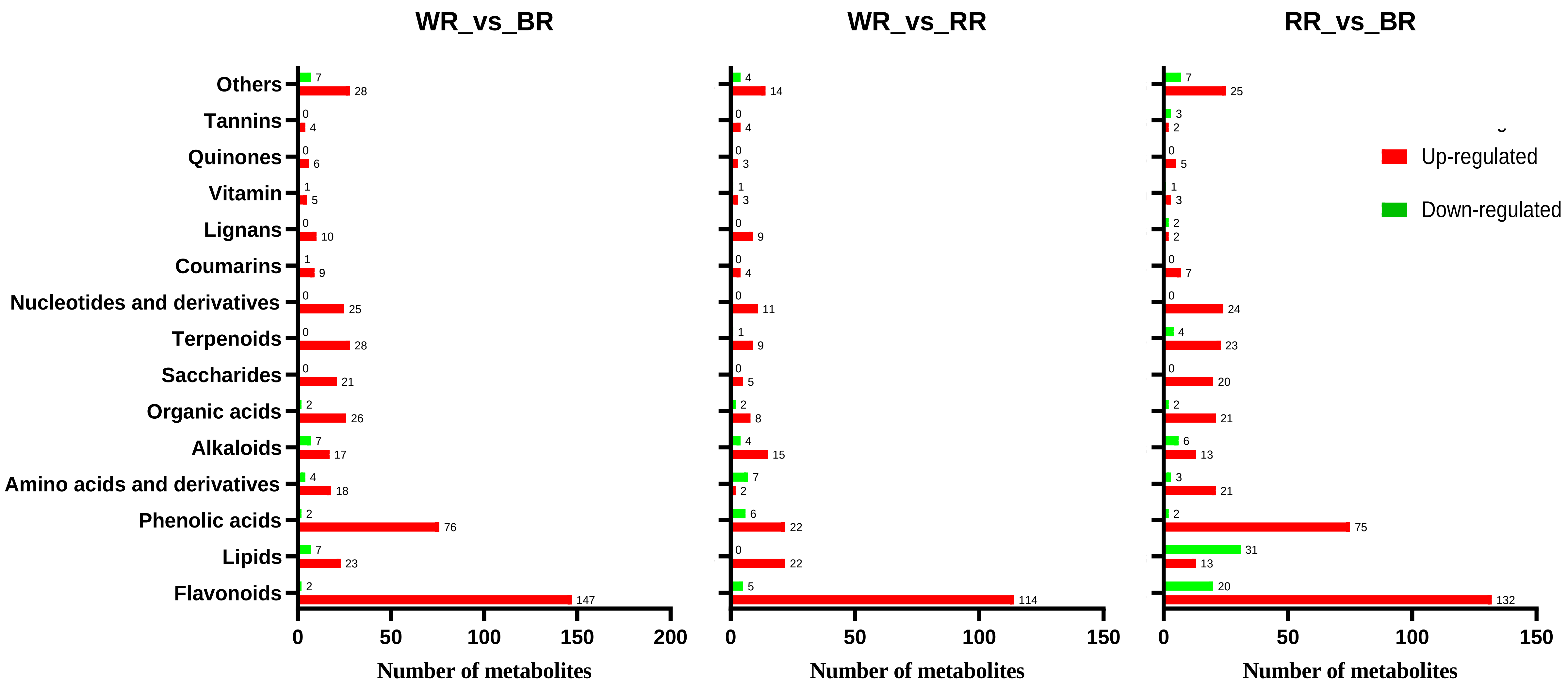

Fig. S6. Classification of the DAMs in other pairwise comparisons. Up-regulation for "X.vs.Y" indicates the metabolite has higher relative content in Ythe WR. BR, black rice; RR, red rice; GR, green rice; WR, white rice.

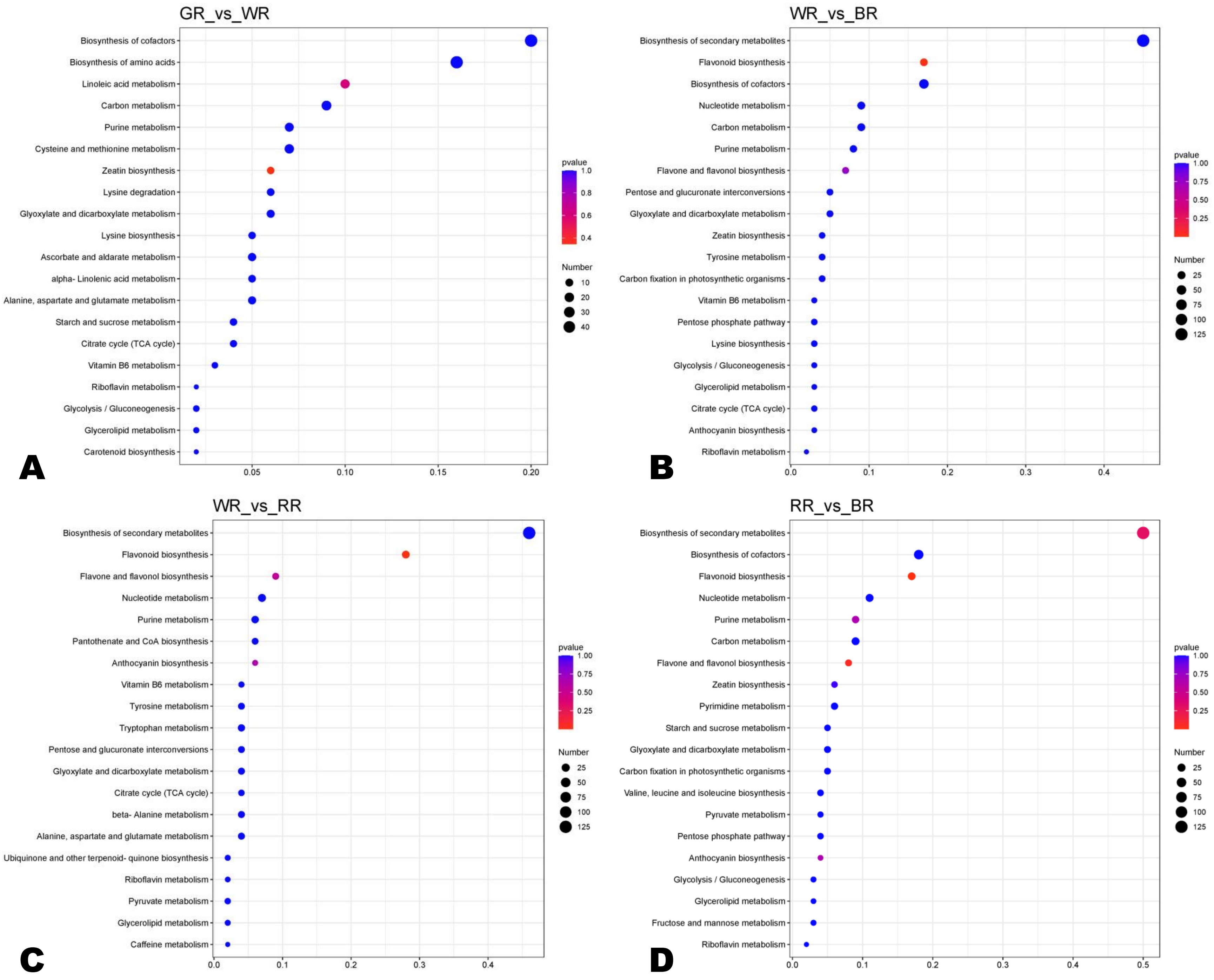

Fig. S7. (A)-(D) KEGG annotation and enrichment results of DAMs in pairwise comparisons between GR_vs_WR, WR_vs_BR, WR_vs_RR and RR_vs_BR, respectively. BR, black rice; RR, red rice; GR, green rice; WR, white rice.

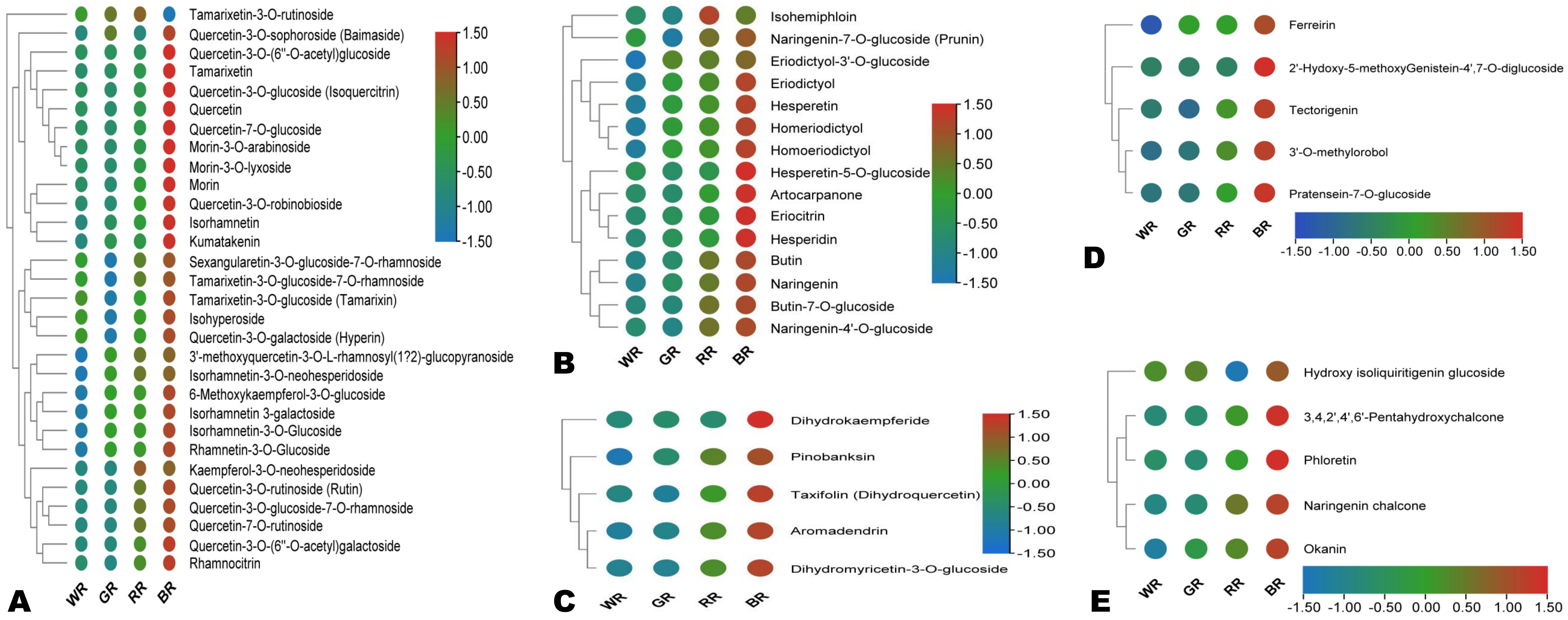

Fig. S8. Variation of other major differentially accumulated bioactive flavonoids in the four rice varieties. (A) Flavonols; (B) Flavannones; (C) Flavanonols; (D) Isoflavones; (E) Chalcones. BR, black rice; RR, red rice; GR, green rice; WR, white rice.

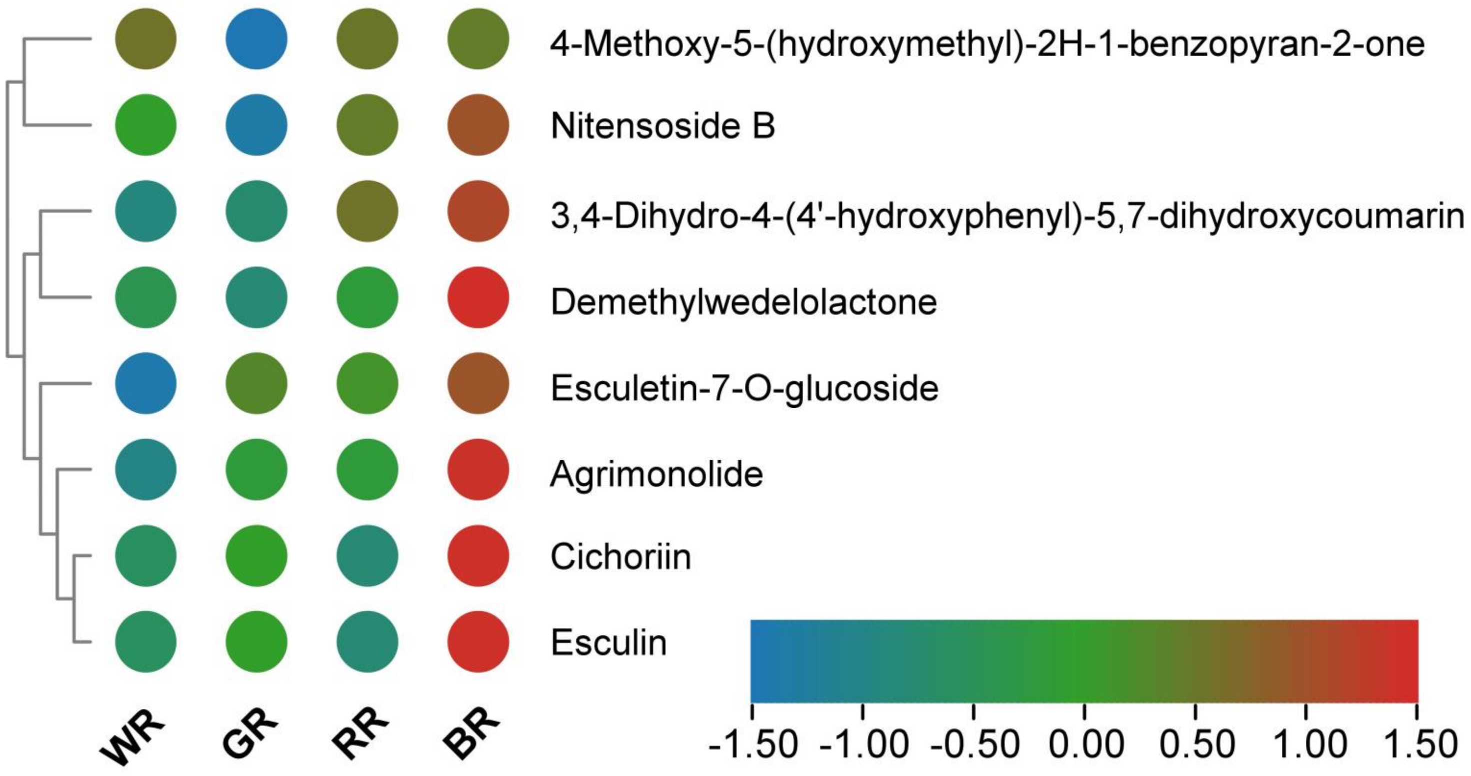

Fig. S9. Variation of major differentially accumulated bioactive coumarins in the four different colored rice grains. BR, black rice; RR, red rice; GR, green rice; WR, white rice.

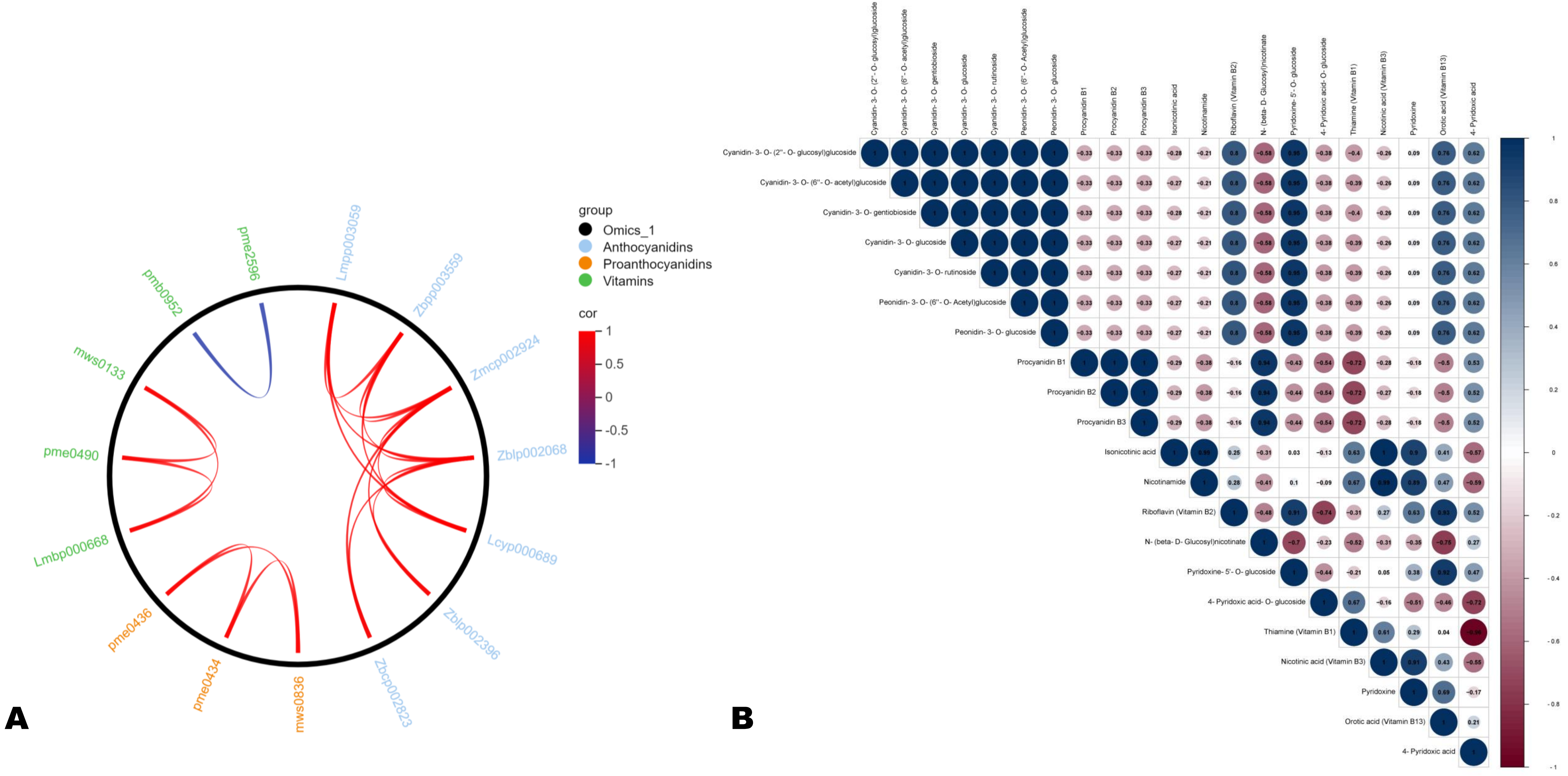

Fig. S10. (A) and (B) Correlations between vitamins and major proanthocyanidins and anthocyanidins. BR, black rice; RR, red rice; GR, green rice; WR, white rice.
